# Self-control mediates the relationship between time perspective and mobile phone addiction in Chinese college students

**DOI:** 10.7717/peerj.16467

**Published:** 2023-11-20

**Authors:** Weigang Pan, Yingzhi Ma, Yihong Long, Ying Wang, Yujie Zhao

**Affiliations:** 1 Laboratory of Emotion and Mental Health, Chongqing University of Arts and Sciences, Chongqing, China; 2 School of Public Administration, South China University of Technology, Guangzhou, China; 3 College of National Culture and Cognitive Science, Guizhou Minzu University, Guiyang, China

**Keywords:** Time perspective, Mobile phone addiction, Self-control, Mediating effect

## Abstract

**Background:**

Mobile phone addiction (MPA) is a prevalent problem among college students, and Chinese college students are a high-risk group for smartphone addiction. MPA has a negative impact on the physical and mental health and academic performance of college students. Studies have explored the influence of many factors on MPA, such as the characteristics of the smartphone itself, the characteristics of the smartphone user, and the environment. However, to date, no studies have explored the protective and risk factors for MPA from the perspective of personality traits. From this perspective, this study explored the influence of time perspective and trait self-control on MPA to identify effective measures to prevent and intervene in MPA in college students.

**Methods:**

The participants in this cross-sectional study were 526 Chinese college students. They completed the Zimbardo Time Perspective Inventory, the Self-Control Scale and the Mobile Phone Addiction Tendency Scale using an online questionnaire tool. Pearson correlation analysis was used to explore the relationships among time perspective, self-control and MPA. A latent variable mediation analysis of the structural equation model was used to examine the mediating role of self-control in the relationship between time perspective and MPA.

**Results:**

Various dimensions of time perspective were strongly associated with MPA. Among these dimensions, past negative (*r* = 0.397, *p* < 0.001), present hedonistic (*r* = 0.207, *p* < 0.001), and present fatalistic perspectives (*r* = 0.444, *p* < 0.001) were positively associated with MPA, while a future time perspective (*r* = −0.200, *p* < 0.001) was negatively associated with MPA. Mediation effects analysis showed that past negative (*β* = 0.034, *p* < 0.001, 95% CI [0.020–0.051]), present hedonistic (*β* = 0.038, *p* < 0.001, 95% CI [0.02–0.06]), present fatalistic (*β* = 0.047, *p* < 0.001, 95% CI [0.031–0.065]) and future orientation perspectives (*β* = −0.093, *p* < 0.001, 95% CI [−0.123–0.069]) indirectly influenced MPA through the mediating effect of self-control.

**Conclusion:**

This study confirmed that a future time perspective and self-control are protective factors for MPA and that past negative, present hedonistic and present fatalistic perspectives are risk factors for MPA. College educators can prevent MPA in college students directly by cultivating their self-control ability, as well as indirectly by increasing their use of future time perspective and reducing past negative, present fatalistic and present hedonistic perspectives.

## Introduction

In recent years, with the development of the internet, the popularity of smartphones has increased. Smartphones have become an indispensable tool in people’s lives and work. However, while smartphones bring much convenience to people, they also cause many negative issues. Problematic smartphone use is emerging as an important public health challenge, as it causes many health problems (Elhai et al., 2016). A recent study of 33,831 participants from 24 countries found that problematic smartphone use is increasing globally, with China, Saudi Arabia, and Malaysia experiencing the most severe cases (Olson et al., 2022). According to the 51st statistical report on the development of China’s internet network released by the China Internet Network Information Center (CNNIC) on March 2, 2023, by December 2022, the number of internet users in China had reached 1.067 billion, with 99.8% of internet users using smartphones to access the internet. Chinese netizens spend an average of 26.7 h per week online (CNNIC, 2023). A systematic literature review found that highly educated people are more likely to develop problematic smartphone use (Busch & McCarthy, 2021). College students are at high risk of smartphone addiction (Haug et al., 2015; Karki et al., 2020; Randjelovic et al., 2021). Mobile phone addiction (MPA) is defined as the compulsive usage of smartphones, which refers to the need for individuals to carry their smartphones with them and check them frequently (Lepp, Li & Barkley, 2016). Some researchers also refer to MPA as the problematic use of smartphones, understood as uncontrolled and excessive use of a mobile phone (Pivetta et al., 2019). A recent study found that the prevalence of MPA among Chinese college students was 36.6% (Mei et al., 2022). Given the large number of college students in China, the number of college students who are prone to MPA should not be underestimated.

MPA can have many negative impacts on college students’ physical and mental health and studies. Studies have found that MPA causes neck and shoulder pain (Alshahrani et al., 2021), reduced physical activity (Kim, Kim & Jee, 2015), sleep deprivation (Chatterjee & Kar, 2021) and other physical problems in users. Additionally, MPA is closely linked to mental health problems such as anxiety (Park, 2019), depression (Grant, Lust & Chamberlain, 2019), negative affect (Wolniewicz et al., 2018), risky behavior (Grant, Lust & Chamberlain, 2019) and decreased subjective well-being (Horwood & Anglim, 2019). Severe MPA may also induce suicidal ideation (Arrivillaga, Rey & Extremera, 2020; Hu et al., 2022). In addition, many studies have found that MPA can cause a decline in college students’ academic performance (Hawi & Samaha, 2016; Kates, Wu & Coryn, 2018; Sunday, Adesope & Maarhuis, 2021).

MPA is affected by many factors, including the characteristics of the smartphone itself, the characteristics of the smartphone user and environmental factors. According to the anonymity, convenience, and escape (ACE) model of internet addiction proposed by Young et al. (2000), the characteristics of the internet itself tend to lead to user addiction. In addition, the psychological states of smartphone users (*e.g.*, boredom proneness (Yang et al., 2020), satisfaction (Hsiao, Chang & Tang, 2016), and motivation (Wang et al., 2015a; Wang et al., 2015b)) can predict MPA. According to compensatory internet use theory, people tend to indulge in internet use to relieve anxiety or escape from real-life problems (Kardefelt-Winther, 2014). Positive personality traits (such as openness and conscientiousness (Cocoradă et al., 2018) and resilience (Robertson, Yan & Rapoza, 2018)) are protective factors for MPA. Negative personality traits, such as impulsivity (Fowler, Gullo & Elphinston, 2020), are risk factors for MPA. Family factors are important variables affecting students’ MPA. Studies have shown that family environment(domestic violence or parental substance addiction) (Kim et al., 2018), negative parenting styles (*e.g.*, parental rejection or overprotection) (Lian et al., 2016) and parental phubbing behavior are positively correlated with teenagers’ mobile phone dependence (Zhang, Ding & Wang, 2021).

For Chinese college students, other factors, such as cultural factors, may need to be included in the discussion. Chinese people are deeply influenced by Confucian culture, which emphasizes self-discipline, self-denial, cultivating character by restraining one’s words and deeds (Duan et al., 2022), and achieving will and moral behavior (Zhang, 2000). Individuals overcome the temptation to use smartphone applications and the influence of external environments through self-discipline, and self-control may be an important safeguard to achieve this goal. In addition, an individual’s cognition, experience and action tendency toward time will affect their value judgment of short-term and long-term goals and thus affect their implementation of self-control behaviors (Dreves & Blackhart, 2019). For example, future-oriented individuals are capable of constant self-regulation and self-control of their behavior and are willing to forgo immediate gratification for distant outcomes of greater value (Przepiorka & Blachnio, 2016). A survey on cultural attitudes toward time showed that Chinese people tend to focus on the future rather than the past (Gu, Zheng & Swerts, 2019). This suggests that time perspective and self-control may be the key personality factors that affect Chinese college students’ MPA. To date, there are no studies on the protective and risk factors for MPA based on personality traits. From this perspective, this study explored the influence of two important personality traits, time perspective and self-control, on college students’ MPA to identify effective measures to prevent and intervene in MPA in college students.

Time perspective is a relatively stable personality trait that reflects people’s consistent views of the past, present, and future, and its dimensions include the past negative, past positive, present hedonistic, present fatalistic and future perspectives (Zimbardo & Boyd, 1999). A past negative perspective reflects an individual’s negative view of the past; a past positive perspective reflects a positive attitude toward the past; a present hedonistic perspective reflects a tendency to enjoy present pleasures without considering future consequences; a present fatalistic perspective reflects a fatalistic, helpless and hopeless attitude toward the future and life; and a future time perspective reflects an individual’s attitude toward achieving future goals. Numerous studies have found that different time perspectives can predict different addictive behaviors. Past negative and present time perspectives are thought to be risk factors for addictive behaviors and are typically associated with substance addictions, such as cigarette (Adams & Nettle, 2009), alcohol (Loose et al., 2019), and drug (Chavarria et al., 2015) addiction, as well as behavioral addictions, such as pathological gambling (Donati et al., 2019) and internet addiction (Chittaro & Vianello, 2013). A future time perspective is considered a protective factor against addictive behavior (Wells et al., 2018). Therefore, helping students to develop a future time perspective can be very beneficial in preventing them from developing addictive behaviors and intervening in their addictive behaviors (Alm & Låftman, 2016).

Self-control is an individual’s ability to modify or suppress dominant responses and thereby regulate his or her thinking, feelings, and behavior to meet the standards of personally set goals (Duckworth & Kern, 2011). High self-control can help individuals maintain healthy eating habits (Haynes, Kemps & Moffitt, 2016) and reduces the risk of substance abuse (Malouf et al., 2014). Low self-control, on the other hand, is a root cause of many social and psychological problems, such as substance abuse (Baumeister & Vonasch, 2015) and impulsive decision-making (Achtziger et al., 2015). A meta-analysis confirmed a significantly negative correlation between students’ self-control and internet addiction (Li et al., 2021). Improving self-control is a very effective way to intervene in and treat addiction (Tang et al., 2015). These studies suggest that preventing and treating MPA by developing students’ self-control is a valuable approach.

Time perspective and self-control are closely related. Zimbardo & Boyd (1999) found that past negative, present fatalistic and present hedonistic perspectives are significantly negatively correlated with self-control, a future time perspective is positively correlated with self-control, and a past positive perspective is not significantly correlated with self-control. Stănescu & Iorga (2015) also confirmed that a future time perspective is positively correlated with self-regulation, past negative and present fatalistic perspectives are negatively correlated with self-regulation, and past positive and present hedonistic perspectives are not significantly correlated with self-control. According to the dual-motive model of self-control, the process of self-control can be regarded as the process of solving dual-motive conflict (Fujita, 2011). Self-control conflicts are conflicts between two motivations: obtaining a smaller reward immediately or pursuing a larger, long-term reward. Time perspective affects self-control by influencing the motivational value of short-term and long-term goals. People with a present hedonistic view of time tend to choose to act on short-term proximal motivations, while those who see things from a future time perspective value long-term motivations more (Dreves & Blackhart, 2019). Baird et al. (2021) found that a future time perspective promotes positive outcomes by affecting target monitoring and self-regulation. These studies suggest that time perspective may indirectly influence students’ MPA through self-control.

**Figure 1:**
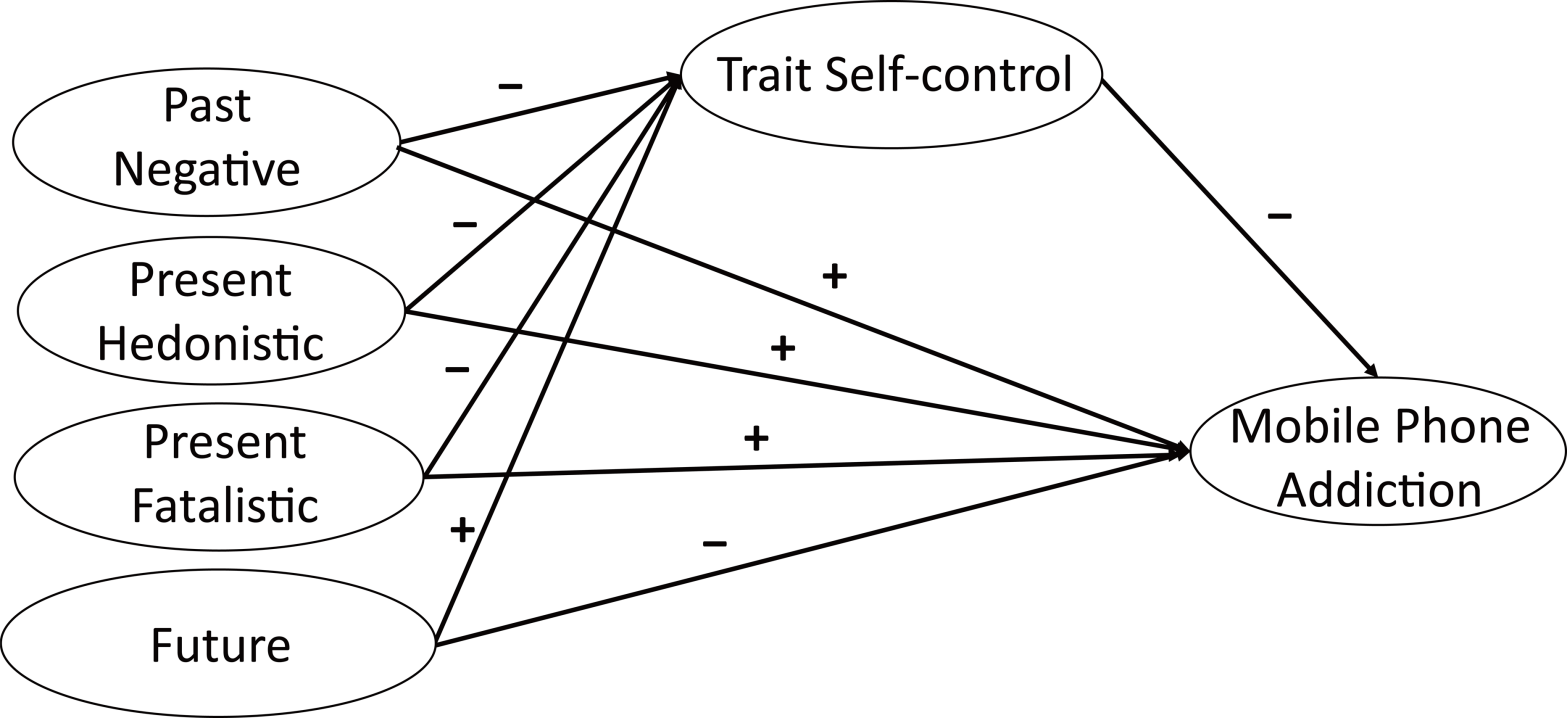
The hypothetical model among variables. Note: +, Positive correlation; −, Negative correlation.

Through the above literature analysis, we found that MPA is a prevalent problem among college students that adversely affects their physical and mental health and academic performance. Many factors affect college students’ MPA. It is important for college educators to discuss the protective and risk factors for MPA from the perspective of college students’ personality traits to reduce their susceptibility to MPA. From this perspective, this study explored the influence of time perspective and trait self-control on MPA in college students to identify effective measures to prevent and intervene in MPA. Based on the existing research results, the following assumptions were made in this study (see Fig. 1):

**Hypothesis 1**: Past negative, present hedonistic, and present fatalistic perspectives positively predict MPA, while a future time perspective negatively predicts MPA.

**Hypothesis 2**: Multiple dimensions of time perspective indirectly influence college students’ MPA through self-control.

## Materials & Methods

### Study design

In this study, a cross-sectional design was used to investigate the relationship among time perspective, self-control and MPA in Chinese college students through three self-rated questionnaires.

### Procedures

First, the research proposal was submitted to the Academic Ethics Committee of Chongqing University of Arts and Sciences for review, and the research was carried out after approval (CQWL-2022-10-8). Then, advertisements were distributed on university campuses to recruit participants, and surveys were conducted using an online questionnaire tool (Questionnaire Star, https://www.wjx.cn/). Before the subjects formally completed the questionnaires, they were informed of the purpose and specific methods of the survey. The questionnaires were conducted anonymously. All subjects needed to provide informed consent online. After the participants completed the questionnaires, they received a gift for their participation.

### Participants

Participants in this study were from three universities in Chongqing, Hubei and Guizhou provinces. All subjects completed the questionnaires within 20 min. A total of 526 college students participated in the questionnaire survey, including 401 female students (76.2%) and 125 male students (23.8%). There were 148 freshmen (28.1%), 154 sophomores (29.3%), 136 juniors (25.9%) and 88 seniors (16.7%). The average age was 19.73 ± 1.37 years old.

### Measures

The Zimbardo Time Perspective Inventory (ZTPI) (Zimbardo & Boyd, 1999) was used to investigate the time perspectives of college students. Originally comprising 56 items, the ZTPI has been revised in several countries, resulting in multiple versions for different cultural backgrounds and groups, each with a different number of questions and content (Orkibi, 2015). In this study, the Chinese version revised by Wang et al. (2015a) was used; it can be applied to measure the time perspectives of Chinese college students. This 20-item scale contains five dimensions: the past positive, past negative, present fatalistic, present hedonistic, and future orientation dimensions. A five-point Likert score is adopted, with responses ranging from 1 (complete disagreement) to 5 (complete agreement). Higher scores indicate a higher time perspective level. The Cronbach’s alpha coefficients of the five dimensions are 0.65 (past positive), 0.71 (past negative), 0.65 (present hedonistic), 0.65 (present fatalistic), and 0.61 (future). In this study, the Cronbach’s alpha coefficients of the five subscales were 0.699, 0.804, 0.554, 0.704 and 0.646, respectively. The reliability of the ZTPI-56 version was found to be better than that of the short version (Przepiorka, Sobol-Kwapinska & Jankowski, 2016). Cortina (1993) pointed out that the smaller the number of items in the scale, the smaller the *α* coefficient will be. Therefore, the *α* coefficient of the ZTPI-56 is generally larger than that of the short scale. However, a scale with too many items will take a long time to complete and place a certain psychological burden on the subjects. Considering that this study required the subjects to complete three scales at the same time, to reduce the workload of the subjects, the short version of the ZTPI was adopted, as done by other researchers (*e.g.*, Orkibi, 2015; Zhang, Howell & Bowerman, 2013). Confirmatory factor analysis of the structural equation model was used to test the factor structure of this version, and the fit indices were obtained (*χ*^2^/*df* = 2.509, *p* < 0.001, Tucker Lewis index (TLI) = 0.886, comparative fit index (CFI) = 0.904, root mean square error of approximation (RMSEA) = 0.054), indicating that the fitting of the five-factor model of this scale was acceptable.

The self-control scale developed by Tangney, Baumeister & Boone (2004) was used to measure trait self-control. The revised Chinese version (Tan & Guo, 2008) can effectively measure the self-control levels of Chinese college students and is scored on a 5-point Likert scale, ranging from complete disagreement (1) to complete agreement (5). Higher scores indicate higher levels of self-control. In the present study, the Cronbach’s *α* coefficient of the total scale was 0.868.

The Mobile Phone Addiction Tendency Scale for college students developed by Xiong et al. (2012) was adopted; it includes four dimensions: withdrawal symptoms (negative physical or psychological effects of not using mobile phones), salience (the use of mobile phones occupies the center of thinking and behavior activities), psychological comfort (the effect of mobile phone use in interpersonal interactions) and mood changes (mood changes caused by mobile phone use). It consists of 16 items scored on a 5-point scale, with scores of 1–5 ranging from complete disagreement to complete agreement. The scores of the four dimensions are summed to obtain the total score of the scale, and the higher the total score is, the more serious the MPA tendency. The scale was shown to provide a good assessment of MPA among Chinese university students (Chen et al., 2016a). In this study, the Cronbach’s *α* coefficient of the total scale was 0.909.

### Data analysis

The data were analyzed with SPSS 22.0 and AMOS 26.0. Pearson correlation analysis was performed to explore the relationships among time perspective, self-control and MPA. The latent variable mediating effect analysis of the structural equation model was used to investigate the mediating role of self-control in the relationship between time perspective and MPA. Since the scales used in this study contain multiple subscales, item parceling was used to simplify the structural equation model. Specifically, the internal-consistency approach (also known as isolated parceling) was used to package items in the same dimension of the scale (Little et al., 2002). *χ*^2^/df, the RMSEA, the CFI and the TLI were used to evaluate the fit of the model in this study. It is generally believed that if *χ*^2^/df is less than 2, the RMSEA is less than 0.08, and the CFI and TLI are more than 0.9, then the model fits well (Hu & Bentler, 1999; Steiger, 1990). Bootstrap tests with 5,000 random samples and 95% confidence intervals were used to analyze the significance of the mediating variable. If the 95% confidence interval did not include 0, the mediation effect was significant (Hayes, 2013).

## Results

### Common method bias

This study used a self-report method to collect data for the three scales, so there may be a common method bias effect. The Harman single-factor test (Fuller et al., 2016) was adopted to test this effect. The results showed that a total of 13 factors had eigenvalues greater than 1, and the variance explained by the first factor was 21.9%, which was less than the critical criterion of 40%. According to the usual criteria (Podsakoff, MacKenzie & Podsakoff, 2012), there was no significant common method bias in this study.

### Descriptive statistics and Pearson correlation analysis

Pearson correlation analysis was used to examine the relationships among time perspective, self-control and MPA. As shown in Table 1, past negative (*r* = 0.397, *p* < 0.001), present hedonistic (*r* = 0.207, *p* < 0.001) and present fatalistic (*r* = 0.444, *p* < 0.001) perspectives were significantly positively correlated with MPA. A future time perspective (*r* =  − 0.200, *p* < 0.001) and self-control (*r* =  − 0.599, *p* < 0.001) were negatively correlated with MPA. In addition, past negative (*r* =  − 0.409, *p* < 0.001), present hedonistic (*r* =  − 0.214, *p* < 0.001) and present fatalistic (*r* =  − 0.465, *p* < 0.001) perspectives were significantly negatively correlated with self-control. In contrast, a future time perspective (*r* = 0.459, *p* < 0.001) was significantly and positively correlated with self-control. Notably, a past positive time perspective was not significantly associated with either self-control (*r* = 0.053, *p* = 0.225) or MPA (*r* =  − 0.066, *p* = 0.13).

**Table 1:** Descriptive statistics and correlation analysis results.

	1	2	3	4	5	6	7
1 Past positive	–						
2 Past negative	−.144^**^^*^	–					
3 Present hedonistic	.277^**^^*^	.144^**^^*^	–				
4 Present fatalistic	−.142^**^^*^	.474^**^^*^	.190^**^^*^	–			
5 Future	.222^**^^*^	−.157^**^^*^	.101^*^	−.250^**^^*^	–		
6 MPA	−0.066	.397^**^^*^	.207^**^^*^	.444^**^^*^	−.200^**^^*^	–	
7 Self-control	0.053	−.409^**^^*^	−.214^**^^*^	−.465^**^^*^	.459^**^^*^	−.599^**^^*^	–
M	15.07	11.14	12.67	10.64	14.85	45.52	60.854
SD	2.814	3.722	2.633	3.114	2.370	11.095	9.8667

**Notes.**

* *p* < 0.05

** *p* < 0.001

*** *p* < 0.001

### The mediating effect of trait self-control on the relationship between time perspective and MPA

**Figure 2:**
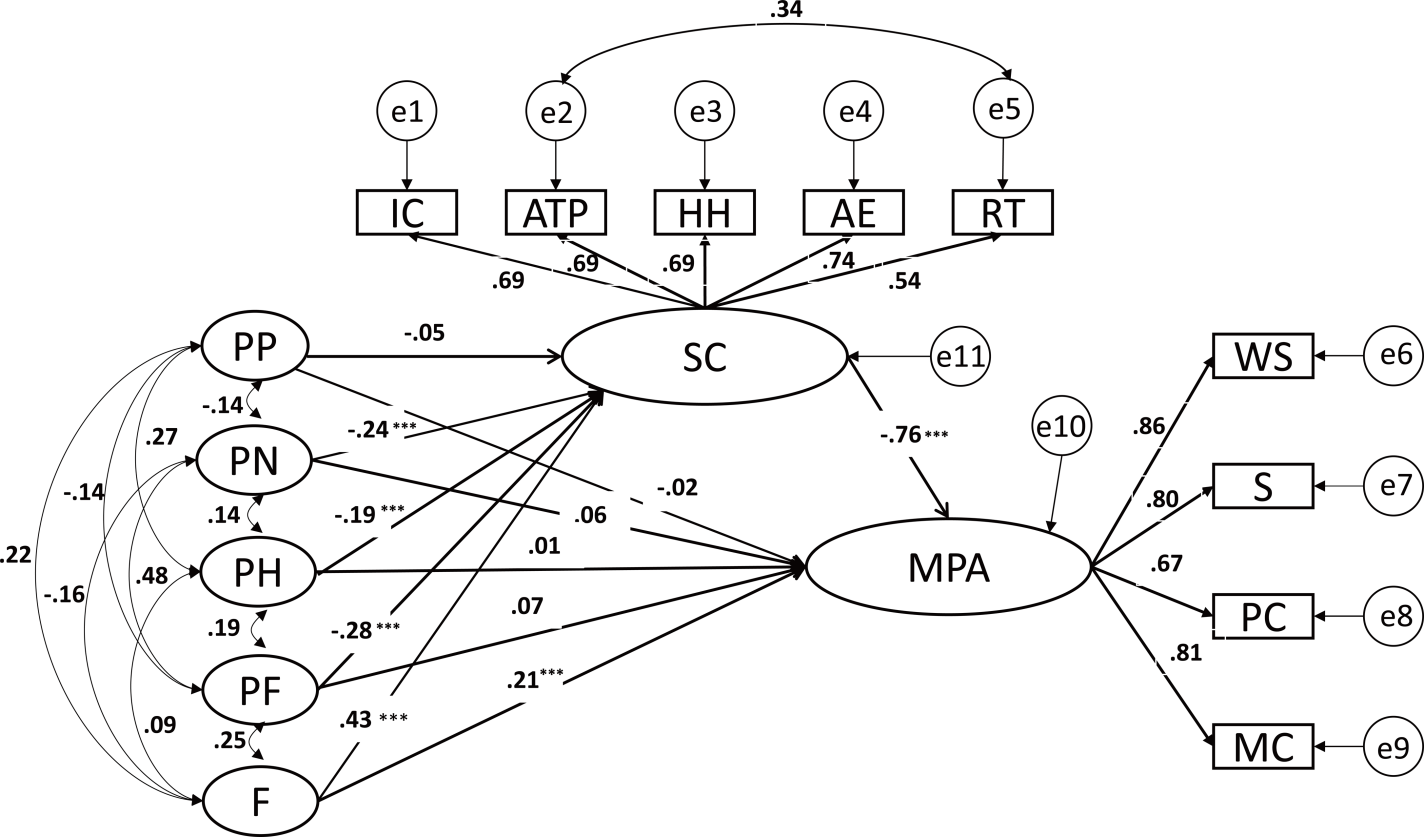
The mediating role of self-control. Note: PP, Past Positive; PN, Past Negative; PH, Present Hedonistic; PF, Present Fatalistic; F, Future; IC, Impulse Control; ATP, Achievement and Task Performance; HH, Healthy Habits; AE, Abstinent Entertainment; RT, Resisting Temptation; SC, Self-control; WS, Withdrawal Symptoms; S, Salience; PC, Psychological Comfort; MC, Mood Changes; MPA, Mobile Phone Addiction. *** *p* < 0.001.

The latent variable mediating effect analysis was used to examine the mediating effect of self-control on the relationship between time perspective and MPA. The five time perspective dimensions were put into the model at the same time to examine the unique influence of each dimension on MPA. The results show that the overall fit of the model was good (*χ*^2^/ *df* = 4.317 (*χ*^2^ = 259.009, *df* = 60), TLI = 0.895, CFI = 0.931, RMSEA = 0.079). The standardization coefficients are shown in Fig. 2. Specifically, the past negative, present hedonistic, present fatalistic and future orientation perspective dimensions were significant predictors of self-control (*β* = −0.240, *p* < 0.001, 95% CI [−0.043 to −0.018]; *β* = −0.192, *p* < 0.001, 95% CI [−0.050 to −0.018]; *β* = −0.277, *p* < 0.001, 95% CI [−0.055 to −0.028]; *β* = 0.431, *p* < 0.001, and 95% CI [0.065–0.099], respectively). Both self-control (*β* = −0.760, *p* < 0.001, 95% CI [−1.425 to −0.899]) and a future time perspective (*β* = 0.210, *p* < 0.001, 95% CI [0.030–0.098]) were significant predictors of MPA. Before the inclusion of self-control as a mediating variable, a future time perspective negatively predicted MPA (*β* = −0.105, t = −2.648, *p* < 0.01). Notably, when self-control was included, the relationship between a future time perspective and MPA reversed.

The mediating effect was tested by the nonparametric percentile bootstrap method with bias correction. The 95% confidence intervals were calculated by repeated random sampling (5000 times). The results (see Table 2) showed that the direct effects of the past negative, present hedonistic and present fatalistic perspectives on MPA were not significant, but the indirect effects of these perspectives on MPA through self-control (*β* = 0.034, *p* < 0.001, 95% CI [0.02–0.051]; *β* = 0.038, *p* < 0.001, 95% CI [0.02–0.06]; *β* = 0.047, *p* < 0.001, 95% CI [0.031–0.065], respectively) and the total effects (*β* = 0.042, *p* < 0.001, 95% CI [0.024–0.06]; *β* = 0.039, *p* < 0.001, 95% CI [0.013–0.063]; *β* = 0.062, *p* < 0.001, 95% CI [0.041–0.083], respectively) were significant. These results suggest that self-control fully mediated the relationships between past negative, present hedonistic, and present fatalistic perspectives and MPA. The direct effect of a future time perspective on MPA (*β* = 0.062, *p* < 0.001, 95% CI [0.03–0.098]) and the indirect effect on MPA through self-control (*β* = −0.093, *p* < 0.001, 95% CI [−0.123 to −0.069]) were both significant, but the total effect was not significant (*β* = −0.031, *p* = 0.053, 95% CI [−0.059–0.001]). Notably, the indirect effect size was approximately 1.5 times that of the direct effect size, but the total effect was smaller than the direct effect, indicating that there was a “suppression effect” (MacKinnon & Lamp, 2021) between a future time perspective and MPA.

**Table 2:** The mediating effects of self-control on time perspective and MPA.

Time perspective	Effects	Estimates	Boot SE	Boot LLCI	Boot ULCI	*p*
PP	PP →SC →MPA	0.009	0.008	−0.006	0.025	0.222
		PP →MPA	−0.010	0.011	−0.034	0.011	0.347
	PP Total effect	−0.001	0.013	−0.026	0.024	0.947
PN	PN →SC→MPA	0.034^***^	0.008	0.020	0.051	0.000
		PN →MPA	0.008	0.009	−0.010	0.026	0.377
	PN Total effect	0.042^***^	0.009	0.024	0.060	0.000
PH	PH →SC →MPA	0.038^***^	0.010	0.020	0.060	0.000
		PH →MPA	0.001	0.013	−0.025	0.025	0.955
	PH Total effect	0.039^**^	0.013	0.013	0.063	0.004
PF	PF →SC →MPA	0.047^***^	0.008	0.031	0.065	0.000
		PF →MPA	0.015	0.011	−0.008	0.037	0.211
	PF Total effect	0.062^**^	0.011	0.041	0.083	0.001
	F →SC →MPA	−0.093^***^	0.014	−0.123	−0.069	0.000
F	F →MPA	0.062^**^	0.017	0.030	0.098	0.000
	F Total effect	−0.031	0.015	−0.059	0.001	0.053

**Notes.**

PP Past Positive PN Past Negative PH Present Hedonistic PF Present Fatalistic F Future SC Self-control MPA Mobile Phone Addiction

The “Effects” column shows indirect effects, direct effects, and total effects in order.

** *p* < 0.01

*** *p* < 0.001

## Discussion

This study explored the influence and mechanism of college students’ time perspectives on MPA and analyzed the mediating role of self-control. The results showed that there was no significant correlation between a past positive perspective and MPA, while past negative, present hedonistic and present fatalistic perspectives were positively correlated with MPA, and a future time perspective was negatively correlated with MPA. The mediating effect analysis showed that self-control mediated the relationships between past negative, present hedonistic, present fatalistic, and future time perspectives and MPA. These findings suggest that past negative, present hedonistic and present fatalistic perspectives are risk factors for MPA, increasing the likelihood of addictive behavior and the difficulty of intervention. Self-control and a future time perspective, on the other hand, are protective factors that reduce the likelihood of addictive behaviors. These results help deepen our understanding of the key factors affecting MPA among college students and provide a theoretical basis for the effective prevention of and intervention in college students’ MPA.

### The relationship between time perspective and MPA

In this study, we found that the a past positive time perspective did not predict MPA, which is consistent with previous findings (Chittaro & Vianello, 2013). This suggests that there is no significant correlation between positive emotions about past events and MPA. In contrast, holding negative attitudes about the past affects mobile phone use. Some studies have found that individuals with a past negative time perspective have worse self-regulation ability and are more likely to experience negative emotions (Davies & Filippopoulos, 2015; Zhang, Ding & Wang, 2021). A past negative time perspective can predict ruminative thinking (Aström et al., 2019), and rumination weakens individuals’ abilities to suppress and transform negative emotions, leading to a decline in their executive function (Yang et al., 2017). Impaired executive function and the inability to resist temptation and control impulses are key causes of addiction (Lechner et al., 2019). Chittaro & Vianello (2013) surveyed 149 Facebook users and found that a past negative perspective could significantly predict pathological internet use.

A present fatalistic view of time involves a fatalistic, helpless, and hopeless attitude toward the future and life (Chen et al., 2016b). A present hedonistic perspective, on the other hand, aims at the near future, focuses only on immediate benefits, and seeks the thrill and pleasure of the moment (Zimbardo, Keough & Boyd, 1997). The tendency to be powerless over the present can lead to the development of negative mental states and ineffective coping strategies, which may promote the emergence of addictive online behavioral dependence (Settanni et al., 2018). Individuals with a present hedonistic time perspective are more prone to risk-taking (Xu et al., 2021). Individuals with present time perspectives (including present fatalistic and present hedonistic perspectives) tend to make quick decisions without planning for the future, achieving immediate gratification without considering the possible consequences of the future (Settanni et al., 2018). According to compensatory internet use theory (Kardefelt-Winther, 2014), individuals with a present time perspective hold a negative attitude toward the future, pursue immediate gratification, and lack the self-control ability to achieve goal-oriented behaviors. Therefore, they are prone to MPA to meet various psychological needs.

This study found a significant negative correlation between a future time perspective and MPA. A future time perspective implies that the pursuit of goals dominated by the behavior is oriented toward the future (Zimbardo & Boyd, 1999). Two aspects of a future time perspective, planning for the future and the realization of future projects, were found to be effective indicators for predicting internet addiction in adults (Przepiorka, Blachnio & Cudo, 2019). Specifically, individuals with a future time perspective have stronger achievement motivations and self-regulation abilities (Baird et al., 2021) and higher incentive values for perceived delayed goals (Teuscher & Mitchell, 2011). Therefore, their corresponding delayed gratification abilities (Kim, Kim & Kim, 2020) and self-control are stronger (Barber et al., 2009), which can reduce the risk of MPA (Han et al., 2017).

### The mediating role of self-control

This study found that the impact of time perspective on MPA is mediated through self-control, which verified Hypothesis 2. Specifically, self-control completely mediated the relationships between past negative, present hedonistic, and present fatalistic perspectives and MPA. Individuals with a past negative time perspective are prone to experience negative emotions (Cao et al., 2019), and negative emotions can impair self-control (Chester et al., 2016). Individuals with a present fatalistic view of time believe that life is determined by fate and is difficult to change; they lack a sense of internal control over the future (Orkibi & Ronen, 2019). Individuals with a present hedonistic view of time display highly impulsive sensation seeking (Muro et al., 2015); they pursue the experience of present stimuli and have defects in delay gratification (Chavarria et al., 2015; Cosenza et al., 2017), which leads to a low level of self-control. In contrast, individuals with a future time perspective tend to think about the possible consequences of the future and engage in rational decision behaviors to obtain long-term benefits (Agarwal & Srivastava, 1981). As a result, they increase the incentive value of distal motivation and decrease the incentive value of proximal motivation, thus increasing their self-control (Dreves & Blackhart, 2019). Many studies have confirmed that the stronger an individual’s self-control is, the lower their risk of MPA (Han et al., 2017; Li et al., 2021). A recent meta-analysis revealed that the positive effects of a future time perspective on individuals are mainly mediated by self-regulation (Baird et al., 2021). The dual system theory of addiction holds that addiction is the result of an imbalance between the reflective system in the prefrontal cortex and the impulsive system in the amygdala, and the reflective system controls the impulsive system through multiple mechanisms after an individual learns social rules (Bechara, 2005). A functional brain imaging study found that core clinical symptoms of addiction include impaired self-control(manifested by impulsivity and compulsion), and symptoms associated with impaired self-control include reduced activity in control networks, including the anterior cingulate, adjacent prefrontal cortex and striatum (Tang et al., 2015). These studies confirm the key role of self-control in the development of MPA.

Interestingly, this study found that a future time perspective negatively predicted MPA before including self-control as the mediating variable, that a suppression effect was generated after introducing the mediating variable, and that the relationship between a future time perspective and MPA was reversed. The indirect effect size of self-control was much larger than the direct effect size of a future time perspective, indicating that a future time perspective and self-control together have a greater impact on MPA. A recent study further confirmed that a high future time perspective combined with high self-control predicted a low level of MPA (Peng et al., 2022). A future time perspective and self-control, while effective in reducing the risk of MPA, may not be beneficial at higher levels. The pursuit of long-term goals in the future while limiting the satisfaction of one’s current needs can have the opposite effect. On the one hand, setting the pursuit goal too far into the future will make individuals perceive that the goal is out of reach, which will have a time discount effect on the value of the future goal (McClure et al., 2007). On the other hand, giving too much thought to the realization of long-term goals at the expense of the satisfaction of current needs can reduce happiness and adversely affect an individual (Wiese et al., 2018). The mediating effect analysis revealed that a future time perspective was inversely associated with MPA, which may be related to these reasons. Clearly, more research is needed in the future to delve deeper into the underlying mechanisms of this phenomenon. These results also suggest the importance of maintaining a reasonable future time perspective level and self-control.

The present study has a few limitations. On the one hand, it was conducted by self-reporting, and subjective reports may have certain measurement biases. For example, the results may have been influenced by the survey environment and the effects of social approval. Future studies should eliminate the influence of these interfering factors by means of objective measurements such as behavioral experiments. It is also possible to use empirical sampling (Conner et al., 2009) to continuously measure the behaviors of the subjects and evaluate their dynamic change processes to understand the real situation of mobile phone use in college students’ daily lives. On the other hand, this study adopted a cross-sectional design and could not examine the causal relationship between variables. Future research should use experimental studies to explore the effects of different levels of time perspective and self-control on MPA. In particular, intervention studies on college students’ time perspectives and self-control should be carried out to investigate the effects of different intervention schemes, providing empirical evidence for follow-up mental health work.

## Conclusions and Practical Implications

This study led to the following conclusions: (1) past negative, present fatalistic and present hedonistic time perspectives are significantly and positively related to MPA as risk factors. A future time perspective and self-control are negatively correlated with MPA as protective factors. (2) Past negative, present fatalistic, present hedonistic and future time perspectives influence MPA through the mediating effect of self-control.

Although there are some limitations in this study, it is the first to explore the risk and protective factors of MPA in Chinese college students from the perspective of personality traits. The results of this study suggest that the prevention of and intervention in MPA in Chinese college students can be initiated by cultivating positive personality traits such as a future time perspective and self-control. A study on the values of Chinese people found that Chinese people attach the most importance to the values of character and self-discipline (Jin, Zheng & Xin, 2009). Therefore, increasing college students’ self-control to reduce the risk of MPA is a direct and effective intervention measure that meets the expectations of the public. The self-control levels of people who are susceptible to MPA can be improved through experimental interventions, and the optimal self-control level needed to effectively prevent MPA can be further explored. In addition, college students can be trained to master effective self-control strategies. It has been found that self-control is not only about resisting temptation and suppressing impulses but also about using effective self-control strategies to achieve goal-oriented behaviors (Katzir et al., 2021). As Sun Tzu said regarding the art of war, we can subdue the army without fighting.

## Supplemental Information

10.7717/peerj.16467/supp-1 Raw Data

## Additional Information and Declarations

### Data Availability

The following information was supplied regarding data availability: The raw measurements were available in the Supplementary File.
